# Focus on “The German TraumaRegister DGU^®^ (TR-DGU)”

**DOI:** 10.1007/s00068-020-01394-1

**Published:** 2020-06-08

**Authors:** Thomas Lustenberger, Rolf Lefering

**Affiliations:** 1 Department of Trauma, Hand and Reconstructive Surgery, Hospital of the Goethe University Frankfurt/Main, Theodor-Stern-Kai 7, 60590 Frankfurt/Main, Germany; 2 University Witten/Herdecke, Cologne, Germany

This focus issue of the *European Journal of Trauma and Emergency Surgery* compiles a collection of outstanding clinical research using the immense dataset of the German TraumaRegister DGU^®^ (TR-DGU). The TR-DGU of the German Trauma Society (Deutsche Gesellschaft für Unfallchirurgie, DGU) was founded in 1993. Currently, approximately 40,000 cases from more than 600 hospitals are entered into the database every year. The selected articles of this focus on issue highlight the immense value the TR-DGU constitutes for the current, but also for the future trauma research.

In the first article by Bieler et al., a broad spectrum of quality indicators for the management of severely injured patients are evaluated [1, 2]. Evaluating the quality of care using such indicators is one of the main tasks of a trauma registry, and therefore the ‘best’ indicators should be considered. Potential quality indicators were selected from various sources, including a literature review, previously used indicators from the TR-DGU, the UK Trauma Audit & Research Network (TARN), the ATLS^®^ manual and the German S3 guidelines on the management of polytraumatized patients. These variables were subsequently assessed by a 12-member interdisciplinary and interprofessional group of experts, using the QUALIFY approach. Out of 43 indicators, assessed by a total of 13 quality criteria, 13 indicators achieved a consensus in at least 9 criteria. These indicators included—among others—time between hospital admission and whole body computed tomography (CT) scan, mortality, administration of tranexamic acid to bleeding patients, use of cranial CT scan in patients with a GCS < 14, time until first emergency surgical intervention, and pre-hospital application of a pelvic sling belt. Further evaluation of these quality indicators will be required prior to implementing them in standardized quality assurance programs.

In the next article of this issue, Wagner and co-authors examine the impact of alcohol on outcome parameters in trauma patients [3]. Previously, numerous clinical and experimental studies found different and even opposing results when it comes to the influence of alcohol on trauma patients’ outcome [4–6]. In the present study by Wagner et al., patients from the TR-DGU with a maximum Abbreviated Injury Scale (MAIS) of 3 or greater were included and were subsequently matched using numerous demographic and clinical variables. While differences between alcohol-positive and -negative patients were found regarding mechanism of injury, sedation rate, and type of transport, no statistically significant differences were noted for outcomes such as the in-hospital complication rate, need for blood transfusion and observed mortality. Further studies are needed to more clearly elucidate the impact of alcohol on patients with varying injury patterns.

Emergency department thoracotomies (EDT) are performed as a salvage procedure for selected patients who arrive in extremis or who arrest shortly before or after arrival. However, the use, indications and associated risks of EDT continue to be debated, mainly because of the low survival rates reported in the literature, particularly in patients suffering blunt trauma [7, 8]. Schulz-Drost and colleagues present in their article an excellent overview of the current use of EDT in European trauma centers contributing data to the TR-DGU [9]. From the 887 patients that underwent an EDT within one hour of arrival to the emergency department, about half of the patients were treated at supra-regional trauma centers. As common in Europe, the vast majority of patients suffered a blunt trauma mechanism (88%). The overall in-hospital mortality, however, did not differ between blunt and penetrating trauma (31% vs. 28%) and approximately 45% of the EDT patients showed a Glasgow Outcome Scale (GOS) of 5 at discharge from hospital, indicating a good recovery. Since many of the previously published data have come from countries where penetrating trauma is predominant, the report by Schulz-Drost et al., which includes mainly blunt injury mechanisms, adds important new information to this field. Most importantly, this study provides much more encouragement for performing EDT for blunt trauma than could be derived from many other papers on this topic.

The analysis by Spering and co-workers addresses another clinically important and timely question [10]. Due to the well-known demographic changes, the number of elderly trauma patients is continuously increasing over the past years. In their TR-DGU analysis, the authors clearly highlight the different injury pattern as well as differences in the clinical management among younger and more elderly (≥ 60 years) trauma patients. Lower intubation rates, less volume replacement in the pre-hospital setting, less air rescue and more restrictive diagnostic imaging for elderly trauma patients are only a few findings that are presented in this study. Therefore, the question whether it is time for a change in the management of severely injured elderly trauma patients is a very valid one and this paper is worthwhile paying attention to and reading.

In the last article of this focus on issue, Relja et al. describe the worldwide unique establishment of a nationwide fluidics biobank of polytraumatized patients, which was initiated by the task force “Network Trauma Research” (Netzwerk-Traumaforschung, NTF) of the German Trauma Society in 2013 [11]. By sampling serum and plasma specimens of severely injured patients at various clinical stages, and complementing the patients clinical data documented in the TR-DGU, this biobank is an outstanding and highly innovative interface allowing for basic, translational as well as clinical research in the future.

It has been a great honor and pleasure to highlight and comment on the valuable contributions to this issue of the *European Journal of Trauma and Emergency Surgery*. The presented articles do not only highlight the tremendous value of the TR-DGU for clinical research, but also give insight into future perspectives of the German TraumaRegister DGU^®^ by complementing clinical data documentation with fluidics samples from severely injured patients.
